# Parkinson-like phenotype in insulin-resistant PED/PEA-15 transgenic mice

**DOI:** 10.1038/srep29967

**Published:** 2016-07-18

**Authors:** Giuseppe Perruolo, Davide Viggiano, Francesca Fiory, Angela Cassese, Cecilia Nigro, Antonietta Liotti, Claudia Miele, Francesco Beguinot, Pietro Formisano

**Affiliations:** 1 URT- Genomica del Diabete, Istituto di Endocrinologia ed Oncologia Sperimentale (IEOS-CNR), Naples, Italy; 2 Dipartimento di Scienze Mediche Traslazionali, Università degli Studi di Napoli “Federico II”, Naples, Italy; 3 Dept Medicine and Health Sciences, Univ. Molise, Italy

## Abstract

Neurological abnormalities, such as Parkinson-like disorders (PlD), are often co-morbidities of Type 2 Diabetic (T2D) patients, although the epidemiological link between these two disorders remains controversial. The PED/PEA-15 protein represents a possible candidate linking T2D and PD, because it is increased in subjects with T2D and is highly expressed in the brain. To test this hypothesis, we have analyzed the neurological and neurochemical phenotype of transgenic mice overexpressing PED/PEA-15 (tgPED). These mice develop impaired glucose tolerance and insulin resistance, accompanied by neurological features resembling PlD: feet clasping, slow and delayed locomotor movements in different behavioral tests in absence of clear cognitive deficits, ataxia or anxiety. Morphological analysis of the brains showed selective modifications of metabolic activity in the striatal region. In the same region, we have observed 26% decrease of dopamine fibers, confirmed by immunohistochemistry and Western Blot for tyrosine hydroxylase. Moreover, they also showed 48% reduction of dopamine levels in the striatum. Thus the tgPED mice may represent a genetic animal model of neurological disease linked to T2D.

PED/PEA-15 is a cytosolic protein enriched in several cells (skeletal muscle, adipocytes, skin fibroblasts and peripheral blood leukocytes) of patients with Type 2 Diabetes (T2D) and their first degree relatives^1^,^2^. Its role in T2D is demonstrated by transgenic mice engineered to overexpress PED/PEA-15 (tgPED), which suffer from abnormal glucose-tolerance, insulin resistance and impaired insulin secretion, with a progression to frank diabetes in animals fed with high fat diet^3^,^4^. PED/PEA-15 works as a molecular adaptor, bound to protein partners involved in apoptosis, survival, growth factor response and glucose metabolism^5^. PED/PEA-15 features a broad pattern of tissue expression, and is particularly enriched in brain^6^. Within the cerebral structures, PED/PEA-15 is distributed in discrete brain areas (hippocampus, pre-frontal cortex, striatum), both in glial and neuronal cells^7^. PED/PEA-15 is also overexpressed in the brain of mice with amyloid deposition and cognitive deficits^8^, thus supporting a link between neurocognitive disorders and metabolic imbalance with features of diabetes mellitus. Indeed, alterations of insulin and IGF-1 signaling have been implicated in the pathogenesis of different neurodegenerative disorders, including Parkinson’s diseases (PD)^9^,^10^. Moreover, T2D has been associated with an increased risk to develop PD in a Finnish population-based survey^11^. Additionally, a recent meta-analysis on 7-population-based cohort studies (1,761,632 individuals) concluded that T2D is associated with increased PD risk by about 38% 12. However, literature data about comorbid diabetes in PD are not yet conclusive. For instance, a metanalysis from case-control studies suggest even an opposite association, with diabetic individuals having a decreased incidence of PD^13^. The contrasting conclusions among observational studies may be due to differences in study design and methodology, and to the difficulty to rule out confounders (such as microvascular damage and diabetic treatment) as risk factors for PD. Interventional studies lend some additional support to the link between T2D and PD: indeed, a reduction in incidence of PD has been observed in T2D patients treated with glitazone, an antidiabetic agent^14^.

Notwithstanding the clinical and therapeutic relevance of this relationship, the molecular mechanism underlying T2D and PD remains largely unknown.

PED/PEA-15 might represent a possible molecular element subserving both T2D and PlD. In fact, several interactors of PED/PEA-15 are expressed in brain and are involved in neuronal physiology and neurodegeneration. Specifically, ERK1/2 and Phospholipase D , both binding partners of PED/PEA-15, are involved in cell death and differentiation in different neuronal systems^15^,^16^. Moreover, the loss-of-function mutations of an interactor of PED/PEA, the serine protease Omi/HtrA2 17, causes PD in humans and motor neuron alterations in murine models^18^. We therefore hypothesize that PED/PEA-15 plays a role in appearance of neurological deficits in patients with T2D. To this aim, we have investigated whether the increased abundance of PED/PEA-15, occurring in mice ubiquitously bearing PED/PEA-15 transgene, may induce motor behavioral abnormalities.

## Results

### General phenotype and locomotor activity of PED/PEA-15 transgenic mice

**Figure 1:**
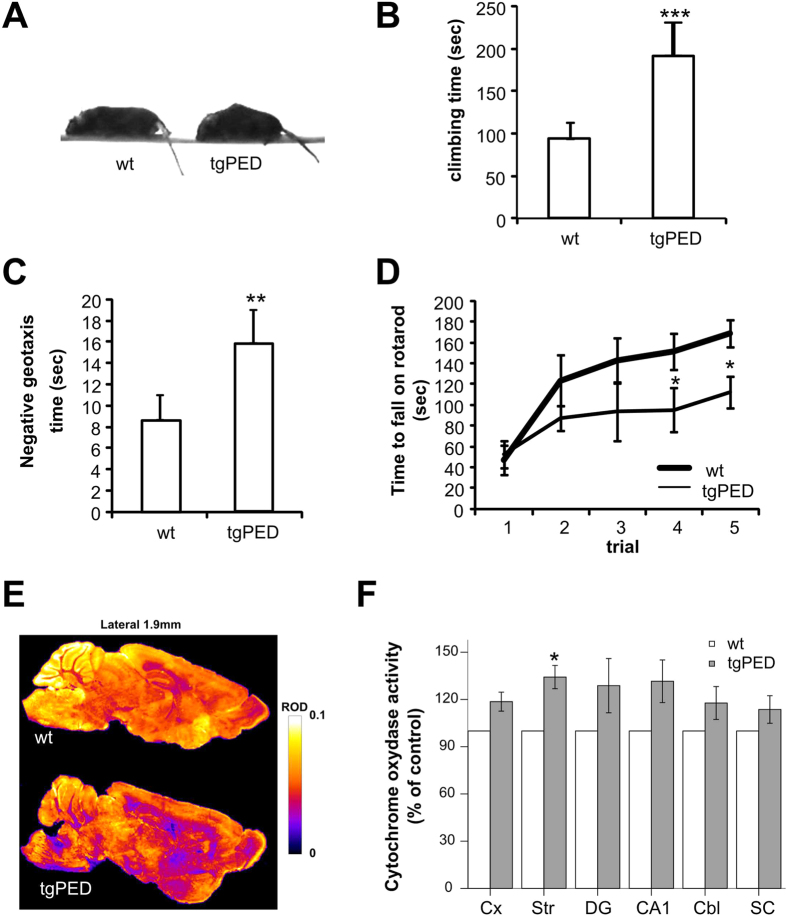
Behavioral and histochemical phenotyping of tgPED mice. (**A)** ‘Aged’ appearance (pronounced hump and grayish coat hair) of tgPED mice compared to wild type littermates. (**B**,**C**) Impaired negative geotaxis indexed as (**B**) the time to reach the top and (**C**) the time to turn around from a head-down position. (**D**) Impaired motor learning ability of tgPED mice. tested using repeated exposures to the rotarod test. (**E**,**F**) modified metabolic activity in the striatum of tgPED mice quantified using the cytochrome oxidase staining on sagittal sections ((**E**) pseudocolor images; (**F**) relative optical density). *p < 0.05 vs. wt mice (t-test for non-paired data).

Transgenic mice over-expressing PED/PEA-15 (tgPED) were viable and fertile with normal litter size, as previously described^3^ and no gross abnormalities of weight and length. Tail elevation and all reflexes were comparable to wild-type littermates (wt) and no tremor was evident. However, at the age of 6 months, all the transgenic mice showed an ”aged” phenotype, as evidenced from the pronounced hump and greyish coat hair, which were not found in wt mice (Fig. 1A). Moreover, when suspended by tail, 85% of tgPED mice revealed retracted hind feet and clenched digit position (feet clasping), whereas all wt mice during tail suspension showed a regular posture with extended hind limbs and digit. TgPED mice did not show forelimb strength modifications compared to wt animals both in the hanging grip test, wire suspension and vertical pole tests (Suppl Table 1).

When tested on an inclined platform, tgPED mice exhibited normal righting response. However, tgPED mice showed two fold increased time to climb (Fig. 1B; Suppl Table 1) and an impaired negative geotaxis, that is a longer time to rotate 180 degrees after placed nose down (Fig. 1C; Suppl Table 1). Similarly, when tested in a Làt maze, transgenic mice showed reduced locomotor activity compared to wt animals, with a decreased horizontal activity indexed as the number of corner crossings in 5 min (Suppl Table 1). At variance, the number of rearings was not significantly changed (Suppl Table 1).

### Stride analysis and anxious behaviour

TgPED mice did not display significant differences in stride length and width compared to the wt mice (Suppl Table 1), thereby lacking gross ataxic signs. This was also confirmed by the beam walking test, which, at the third exposure, did not show significant differences in number of limb displacements between wt and tgPED mice (Suppl Table 1). However, during the first exposure to the beam test, the tgPED mice showed a significant greater number of errors (Suppl Table 1). Moreover, the involvement of vestibular system was also excluded by the normal righting reflex of tgPED animals when placed on their back (none of the animals tested showed absent or slow righting reflex). O maze and elevated plus maze (EPM) were then used to verify if the locomotor hypoactivity was due to greater anxiety. Both in O-maze and in EPM, tgPED and wt mice did not show significant difference in the time spent in unprotected sectors of the mazes (Suppl Table 1). Moreover, in the object recognition task tgPED mice spent more time on the already known object compared to wt mice, and less time on the novel object, which suggests neophobia but not memory impairment (Suppl. Table 1).

### Rotarod test

To test the motor learning, mice were trained on a Rotarod during five consecutive trials. TgPED mice did not show significant differences in time spent on rotating rod compared to wt during the first exposure of animals (Fig. 1D, Suppl Table 1). Both wt and transgenic animals improved their ability to remain on a rotating rod as a function of the number of trials, but motor learning velocity was significantly slower in tgPED mice (Fig. 1D, Suppl Table 1).

### Explicit memory tests

In a typical spatial learning task, the Barnes’ maze, tgPED mice reached the same performance level of control mice, demonstrating a preserved overall learning ability (Trial effect: F_3,36_ = 9.1, p ≪ 0.01, trial * genotype interaction effect: F_3,36_ = 9.1, p = 0.5 , Fig. S1, Suppl Table 1). Similarly, analysis of the pattern of exploration of the maze did not show significant differences between TgPED and wt mice. Indeed, both working memory errors, indexed by the number of holes visited two times in the same session, and procedural errors, indexed by the number of time animals encountered the goal without recognizing it, were not statistically different between the two groups (Suppl Table 1). Moreover, in burrowing test, which requires the integrity of hippocampal system, tgPED did not show difference from wt mice. Finally, in a spontaneous alternation task in a T maze, the number of working memory errors indexed by the exploration of the same arm visited in the previous session was not statistically different in the two groups of animals (Suppl Table 1).

### Brain metabolic activity, Tyrosine Hydroxylase and dopamine levels in Caudate-Putamen (CPu)

In order to identify brain regions involved in these behavioural phenotypes, we mapped brain metabolic activity using cytochrome oxidase histochemistry. TgPED mice showed an overall increased metabolic activity in all brain regions, as shown by two-way ANOVA with genotype and brain regions as factors (F_1,36_ = 9.3, p = 0.004). Nevertheless, this change was significant only in the Striatal region, as shown by multiple post-hoc comparisons (Fig. 1E,F; p = 0.027, t-test for non paired data). Since the CPu is regulated by dopamine system and dopamine (DA) release may change locomotor activity, we further analyzed CPu region using Tyrosine Hydroxylase (TH) and DA immunohistochemistry.

**Figure 2:**
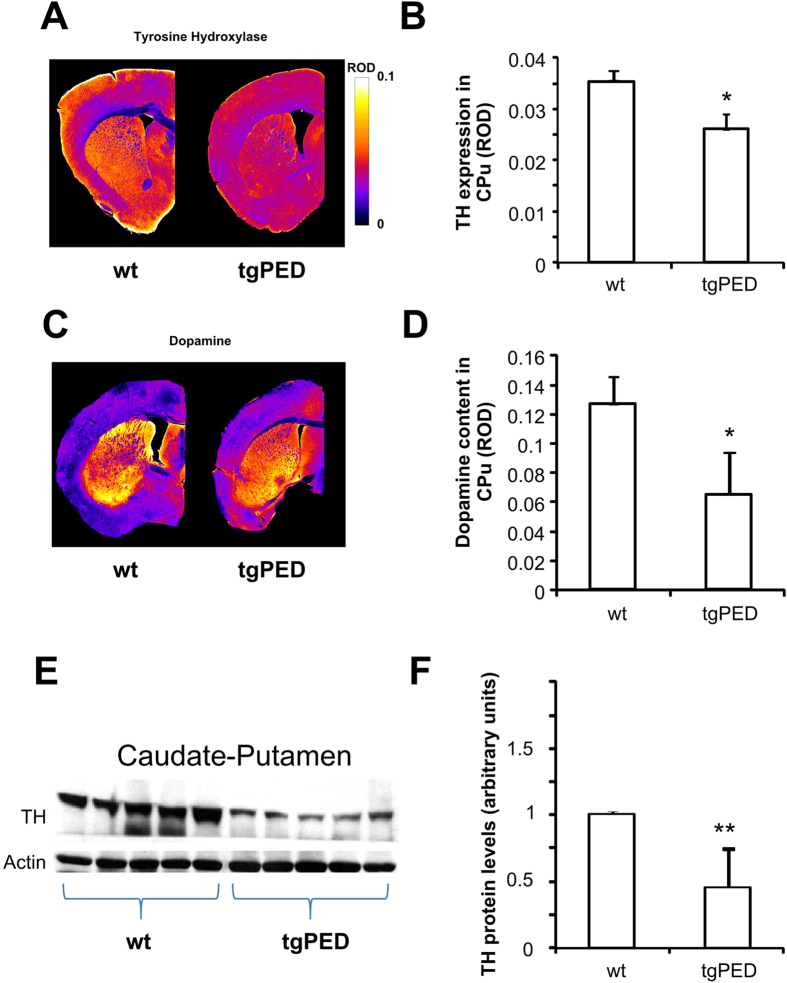
Decreased tyrosine hydroxylase and dopamine content in the striatum of tgPED mice. (**A**,**B**) TH staining of the striatum of Wt and tgPED mice in coronal sections (**A**) pseudocolor images, (**B)** quantification of the relative optical density of the striatum). (**C**,**D**) Dopamine immunohistochemistry in the striatum of Wt and tgPED mice in adjacent coronal sections ((**C**) pseudocolor images, (**D**) quantification of the relative optical density of the striatum). (**E**) Total protein lysates from brain homogenates were analyzed by Western blot with anti-TH antibody. Actin was used as loading control. (**E**) representative experiment is shown. (**F**) Bands were scanned, densitometrically quantitated using NIH Image J software and the resulting data were plotted as bar graph. Values represent the mean ± SD of three independent experiments. *p < 0.05 vs. wt mice; **p < 0.005 (t-test for non-paired data).

Quantitative morphometric analysis for TH staining showed a 26% decrease in the CPu (Fig. 2A,B). This was paralleled by a 48% reduction in DA levels (Fig. 2C,D). Similarly, TH protein (Fig. 2E,F) was reduced in CPu extracts of tgPED mice compared to their wild-type littermates. It should also be noticed that acetylcholine (Ach), another local neurotransmitter with neuromodulatory activity on the CPu, did not show significant changes in the CPu of Tg mice, based on acetylcholinesterase histochemistry as a marker (data not shown).

## Discussion

The main observation of the present study is that the overexpression of PED/PEA-15, which induces impaired glucose tolerance, is accompanied by hypodopaminergic phenotype and hypoactivity, resembling the phenotype of several animal models with Parkinson-like symtoms. Indeed, hypoactivity can be the result of dopamine system hypofunction^19^,^20^,^21^.

The hypoactive behaviour is a constant neurological feature of many animal models of PlD, as well as mice with inactivation of TH in DA neurons (dopamine deficient mice)^21^.

This behavior likely parallels the hypokinesia present in human PlD. Accordingly, the slower learning ability on Rotarod of tgPED mice can be linked to the amount of dopamine, as occurs in other hypodopaminergic animal models^22^,^23^. However, based on rotarod data, we cannot exclude the effect of fatigue, even in the presence of normal muscular strength at the hand grip and normal muscular resistance at the hanging wire test.

In addition, TgPED mice also show later latency in negative geotaxis, which resembles the difficulty to start movements in PlD patients.

Indeed, the hypoactivity of tgPED mice was paralleled by lower dopamine content and an increased metabolic activity in the CPu. This phenotype (locomotor hypoactivity + hypodopaminergic + increased CPu metabolism) has been already observed in another PlD model, the Girk2Wv (weaver) mice^24^.

TgPED mice also show foot clasping, a symptom reported in several other transgenic mice bearing different neurological alterations such as mice deficient for Phospholipase A2 25, Nuclear factor IA^26^, CREB^27^, Junctophilin^28^, HD gene gene with consequent striatal dysfunction^29^, SCA3 mice^30^, pPS1-A246E mutation^31^, Caveolin^32^, Npas3 33, mGAT1^34^ and mice over expressing BMP4 35. Due to largely different behavioral phenotype of these animals, foot clasping should be considered as a rather aspecific neurological symptom.

The kyphosis observed in tgPED mice should also be regarded as an unspecific symptom, although it has been observed in another PlD mouse model, the parkin-deficient mice.

tgPED animals did not show significant differences in anxiety. Unfortunately, the variability in the two groups was high, thereby making difficult the complete exclusion of an “anxious” phenotype. The hypokinesia cannot be easily disentangled from anxiety, in animal models, since anxious tests require, in turn, locomotor activity. Indeed, the total activity of tgPED mice in the EPM was significantly lower compared to wt mice, and this may further confound the interpretation of data (see Suppl Table 1).

Similarly, our model did not show learning and memory deficits (spatial learning, working memory), nor gait modifications compared to wt.

These four symptoms (reduced motor activity, anxiety, memory impairment and gait modifications) are usually not simultaneously found in animal models of PlD: for instance, in genetic models of PD, the behavioral phenotype is rather variable (see Suppl Table 2).

At variance, tgPED mice show a hypo-dopaminergic profile and a resulting hypo-locomotion, without evident modifications of memory and gait . These observations point out that the phenotype in tgPED mice is mild, and possibly additional genetic/environmental factors are needed to give a full PD phenotype.

It is already known that suppression of PED/PEA-15 brain expression is accompanied by behavioural impairment and neuroprotection^36^,^37^, whereas no data are available about its overexpression, which leads to impaired glucose tolerance. Preliminary data from our group suggest that PED/PEA-15 gene overexpression is induced by high fat diet in skeletal muscle and after stress. It is possible that the overexpression of PED/PEA-15 modifies the insulin sensitivity in the brain^3^, which might be partially responsible for dopaminergic sensitivity to degeneration. Therefore it is worth to further characterize the mechanism by which PED/PEA-15 overexpression modulates the dopamine system.

These considerations foster some important translational implication (pathogenetic, diagnostic and therapeutical). First, on the pathogenetic side, it would be tempting to speculate that the origin of PlD in T2D is not entirely due to the hyperglycemic/vascular state. It is actually possible that the dysregulation of certain molecular targets, such as PED/PEA-15, may lead to parallel and independent damages in systemic glucose metabolism and brain metabolism. Our model, in fact, does show behavioral modifications even before true hyperglycaemia is present. Second, it is possible that PlD emerges when only glucose intolerance is present, in absence of diabetes. If this conclusion holds true, one can expect even a stronger connection between dysmetabolism and PD, because glucose intolerance is rarely tested in PD patients. Finally, it is plausible that in T2D-linked PlD the therapeutic approach should not be limited to the hyperglycemic state, because brain damage could be due to a hyperglycemia-independent mechanism. However, it could be interesting to test drugs improving insulin sensitivity in the brain in a subset of PlD patients, as a potential tool for PD prevention.

## Methods

### Behavioral monitoring in transgenic mice overexpressing PED/PEA

Generation of mice overexpressing PED/PEA -15 (tgPED) has been described elsewhere^3^. The animals used in this study have been characterized as indicated in Table 1.

Behavioral monitoring was performed on male tgPED (n = 11) and Wt littermates (n = 11), at the age of 6 months, during the light phase between 10:00 a.m. – 17:00 p.m. A modified SHIRPA protocol has been used as a first screening to observe gross abnormalities in posture and sensorial response. The presence of foot clasping (abduction of hindlimbs) has been evaluated suspending mice by their tails. Subsequently, mice have been tested for their negative geotaxis, stride pattern, motor abilities and memory; less stressful tests (geotaxis, stride analysis) were conducted first in the same experimental session; a minimum of one day separated the testing sessions.

All animal procedures were approved by the ethical committee of the University of Naples “Federico II” and comply with the standards of the European Union.

### Negative geotaxis and climbing

Mice were placed on the lower part of an inclined grid (13 cm wide, 50 cm high, 60° inclination, 12 mm mesh, borders covered by tape), facing the floor. The time to turn (negative geotaxis) and to reach the top of the grid was recorded, with a maximum time of 5 min.

### Rotarod and motor learning rate

Evaluation of motor coordination was conducted using a standard mouse rotarod (UgoBasilesrl, Varese Italy) measuring the time a mouse remained on a rotating rod. The rubber rotating rod (diameter 7 cm, length 11 cm, raised 35 cm above the bottom of the rotarod enclosure) rotated at increasing speed from 4 to 40 rpm in 5 min. Mice were allowed to stay on the rotating rod for a maximum of 5 min for 5 successive trials. Trials were separated by at least 60 s. The latency to fall was recorded for each trial, and improvements from trial to trial was used as an index of motor learning rate.

### Footprint analysis, beam walking, O-maze and elevated plus maze, object recognition task, Barnes’ circular maze and spontaneous alternation

See Supplementary informations.

Forelimb force has been tested with three tests : (i) hang grip force test, (ii) wire hang test and (iii) vertical pole test (see supplementary methods for details).

### Morphological analysis and immunohistochemistry

Mice were trans-cardiacally perfused with 4% paraformaldehyde in 0.1 M phosphate buffer (pH 7.4) and post fixed in the same fixative at 4 °C for 2 h. After cryoprotection and freezing, 20 μ thick cryosections were mounted on SuperFrost Plus slides and stored at -80. For dopamine immunohistochemistry, mice were perfused with the addition of Sodium meta-bisulphite in the perfusion buffer, to prevent oxidation of dopamine. Basal metabolic activity of different brain regions was evaluated by cytochrome oxydase (COX) staining. Sections have been incubated for 2 h at room temperature with the following staining medium: Cytochrome C 0.04% (Sigma-Aldrich), Sucrose 5%, DMSO 0.2%, DAB 0.05% in MOPS 0.1 M pH7.4. All brains have been stained at the same time in the same staining solution to minimize variability due to the staining procedure.

Immunohistochemistry for tyrosine hydroxylase (TH) was performed as follows: after rinsing in PBS, slides were incubated with a mouse monoclonal anti- TH (1:500, Sigma) or with anti-dopamine (1:100 with the addition of sodium metabisulphite) in PBS overnight with shaking at + 4 °C. To allow optimal homogeneous staining among silces, the HybriWell system has been used (BioScience). After three rinses in PBS, sections were then incubated with anti-mouse biotinylated antibody (1:100, Vector) for 1 hour at room temperature with shaking, and the ABC MOM system (Vector) used to visualize the reaction product (prepared according the supplier’s instructions). The reaction was visualized using DAB as chromogen. Slides have been analysed with a Zeiss Axioskop 20, equipped with a CCD camera (Hamamatsu Photonics, Italy, C5405) and motorized XYZ stage (Proscan II, Prior). The entire brain sections have been acquired using tiled fields from 10x objective (MCID Elite; Imaging Res. Inc., Canada). At least 10 slices per brain have been imaged for each staining technique. Markers expression levels have been quantified as relative optical density (ROD = log (256/grey level). ROD units, after background subtraction, are correlated to the antigen concentration.

### Western Blot Analysis

Mice were euthanized and brain samples were collected rapidly, snap-frozen in liquid nitrogen and stored at −80 °C. Tissue samples were homogenized in a Polytron Homogenizer (Brinkmann Instruments, NY, USA) in 20 ml T-PER reagent (g tissue)^−1^ according to the manufacturer’s instructions (Pierce, IL, USA). Tissues homogenates were then separated by SDS-PAGE and analyzed by Western blot as previously described.

### Statistics

Data have been analyzed using Excel and SPSS software (SPSS Inc. Released 2004. SPSS for Windows, Version 13.0). Comparisons among groups have been analyzed using Student’s t-test for non paired data in case of continuous variables and Mann Whithey U test in case of discrete variables. Rotarod and Barnes’ maze data have been analyzed by two way ANOVA using training session and genotype as factors. Cytochrome oxidase activity levels has been analyzed with two way ANOVA using brain region and genotype as factors. Data are expressed as mean ± SEM. Rejection level of the null hypothesis has been set to p < 0.05.

## Additional Information

**How to cite this article**: Perruolo, G. *et al*. Parkinson-like phenotype in insulin-resistant PED/PEA-15 transgenic mice. *Sci. Rep*. **6**, 29967; doi: 10.1038/srep29967 (2016).

## Supplementary Material

Supplementary Information

## Figures and Tables

**Table 1:** Metabolic characteristics of TgPed/Pea-15 mice.

Variable	WT mice	Tg-Ped mice	Test t
Body weight (g)	26.6 ± 1.6	25.9 ± 1.7	n.s.
Fed glycaemia (mg/dl)	139 ± 21	164 ± 125	p < 0.01
GTT AUC (mg/dl·min-1)	9.1 ± 1.9	17.5 ± 1.4	p < 0.001
ITT AUC (mg/dl·min-1)	7.9 ± 1.1	11.7 ± 1.8	p < 0.001

Glucose levels were measured by a portable glucometer (One Touch Ultra; Johnson & Johnson), from blood collected by tail vein. Glucose and insulin tolerance tests (GTT and ITT) were performed in fasting and random fed mice, respectively, by measuring blood glucose at different times (0, 15, 30, 45, 60, 90 and 120 minutes) after an intraperitoneal injection of glucose (2 mg/kg) or insulin (0.75 U/g), as described in Vigliotta *et al*.^3^. Glucose and insulin tolerance values are reported as the area under the curves (AUC) of glycaemic levels. Statistical analysis was evaluated by the Student’s t-test.
