# Supplementary material for: Parkinson-like phenotype in insulin-resistant PED/PEA-15 transgenic mice
Source: Sci Rep. 2016 Jul 18;6:29967. doi: 10.1038/srep29967 (PMC4947959; doi:10.1038/srep29967)
Supplement: Supplementary Information [file srep29967-s1.pdf]

Parkinson-like phenotype in insulin-resistant PED/PEA-15 transgenic mice

Giuseppe Perruolo, Davide Viggiano, Francesca Fiory, Angela Cassese, Cecilia Nigro, Antonietta Liotti, Claudia Miele, Francesco Beguinot, Pietro Formisano

Supplementary Methods

Behavioral tests

Footprint analysis. Animals have been required to walk in a 50cm long, 5cm wide, alley with a white paper covering the floor. Then the hind paws have been dipped in black ink, the animal placed at one end of the alley and let free to walk, repeating three times the procedure. Footprints recorded on the sheet of paper on the floor have been used to determine stride length, stride variability, base of support and limb rotation excluding series of less than 4 sequential steps.

Hanging grip force. The hanging grip force of the forelimb was measured using a dynamometer with a wire bail (diam.1mm) attached to the coil, to serve as gripping place for the mouse. Mice were lifted and held by their tail so that both their forepaws were placed on the thread, immediately inducing a grasping behaviour on the wire. Mice were then gently pulled backward by the tail with their posture parallel to the surface of the table until they released the wire. The peak force exerted by mouse forelimbs as their grip was broken by the experimenter pulling the mouse by the base of the tail from the dynamometer was recorded in Newtons (N). Three trials were performed about 20s apart for each mouse, and the maximal force exerted among the three trials was then used for analysis.

Hang wire test. In the wire hang test the mouse was suspended by its forelimbs in the center of a thread (diam. 2mm, length 20 cm) suspended between two platforms about 20cm from the ground. Latency to fall, which requires optimal balance and grip strength, was recorded, with a 60 s cut-off time.

Vertical pole test. In the vertical pole test the animal was placed in the centre of a wooden dowel (diam. 2cm, length 40 cm) , held in horizontal position. The pole was then gradually lifted to a vertical position so that the mouse was facing up. Latency to fall off the pole was recorded, with a 60 s cut-off time. This test require both normal muscular ability, and motor coordination/ balance.

Burrowing test. The burrowing test has been adapted from a previously published apparatus (Deacon, R. Assessing Burrowing, Nest Construction, and Hoarding in Mice. J. Vis. Exp.(59), e2607, doi:10.3791/2607 (2012)). Briefly, an opaque plastic burrow (diameter: 7cm), filled with 66g of food pellet normally supplied as diet wasplaced overnight in the home cage of individually house mice. The amount of pellet displaced was measured by weighing the burrow before and after the test**.**

Beam walking. Each animal was placed on a 10x10 cm starting platform brightly illuminated, connected to the home cage through a horizontal wooden beam (45cm long, 1.6cm wide). The task was repeated three times and the number of falls (errors) through the beam was counted.

Làt maze. The experimental apparatus is similar to a previously published apparatus (Ruocco L, Viggiano D, Pignatelli M, Iannaccone T, Rimoli MG, Melisi D, et al. Galactosilated dopamine increases attention without reducing activity in C57BL/6 mice. Behav Brain Res. 2008 187:449–54), with minor modifications. Briefly, mice were allowed to explore the square corridor resulting between two squared boxes placed one inside the other (the total length of the corridor was 80 cm, all the walls were made of transparent plexiglas, the floor consisted of a removable white PVC sheet). Mice were allowed to explore the maze for 5 minutes, the behavior was videotaped and the traveled distance and number of rearings were scored off-line.

O-maze and Elevated Plus maze. An elevated O ('null') maze was used, consisting in an annular runway (diam.46cm, width 5.5cm, 40cm above the floor), with two opposing 90° sectors of the runway protected by an inner and outer opaque wall (height 16 cm) and the two remaining sectors unprotected. Animals were allowed to explore for 10min and the percent of time spent in open sectors analysed. The presence of anxious behavior was also confirmed with the elevated plus maze (EPM). The elevated plus-maze consisted of two open and two closed arms of the same size (25 x 5 cm). Each mouse was placed in the central square of the maze, facing one of the closed arms. Animals were allowed to explore for 10min and the percent of time spent in open sectors were analyzed.

Delay-dependent one-trial object recognition (ORT). To test episodic memory, mice have been tested with a one-trial object recognition task. To this aim, after exposure to the arena, mice have been exposed in the same environment to a pair of identical objects (sample trial). After a delay of 24 hours animals have been released again into the open-field, now containing two objects, a familiar one, known from the sample trial, and a novel object. For each mouse, the time spent exploring the objects (in seconds) during the test trial has been scored. **Data are presented as**

recognition index, that is the time spent investigating the novel object relative to the total object investigation.

Barnes circular maze task. To test spatial memory the Barnes' maze has been used, which is a dry version of the Morris water test. This test has several advantages such as to minimize thigmotactic behaviour (no walls are present) and to allow animals to use their normal locomotion (walking, whereas in Morris' maze they are required to swim). Mice have been placed in the middle of a circular platform (diam=1.22m.; elevation from the floor= 40 cm), with 36 equally spaced holes (each 5cm diameter) around the periphery (5 cm from the perimeter). Only one hole led to a dark escape box (5 cm x 5 cm x 11 cm) fixed in relation to the distal environmental cues. The platform surface was brightly illuminated from above as motivation to escape in the dark box. The test lasted 5 min and was repeated for 4 days once a day. The amount of time that the mice took to enter the escape hole (escape latency) and the escape path was recorded.

Spontaneous alternation. Spontaneous alternation is the innate tendency of rodents to alternate free choices in a T-maze over a series of successive runs. The T-maze was made of Plexiglas with a main stem (70cm long x 10 cm wide x 20 cm high) and two arms (30 cm long x 10 cm wide x 20 cm high) positioned at 90° angle relative to the main stem. A start box (15 cm long x 10 cm wide) was separated from the main stem by a sliding door. Cues placed above the arms served as spatial reference points. Mice were free to explore the maze for 9 consecutive trials. At the beginning of a trial, the mouse was placed in the start box for 30sec followed by the opening of the door to the stem. When the mouse entered one of the arms, the door of that arm was then closed. The chosen arm and the elapsed time between the opening of the start box and choice of an arm (choice latency) were recorded. After 30sec confinement in the chosen arm, the mouse was removed and returned to the start box for the next trial. The percentage of alternation over the nine trials was determined for each mouse and used as an index of working memory/attention performance. This percentage was defined as entry in a different arm of the T-maze over successive trials (i.e. left-right-left-right-etc.)

Supplementary figures:

Figure S1: Explicit memory testing in the Barnes' maze. Animals (n=7 per group) have been tested on the Barnes' circular maze for four days as indicated in Supplementary methods, and the time to reach the target hole recorded. Data represent mean ±SEM.

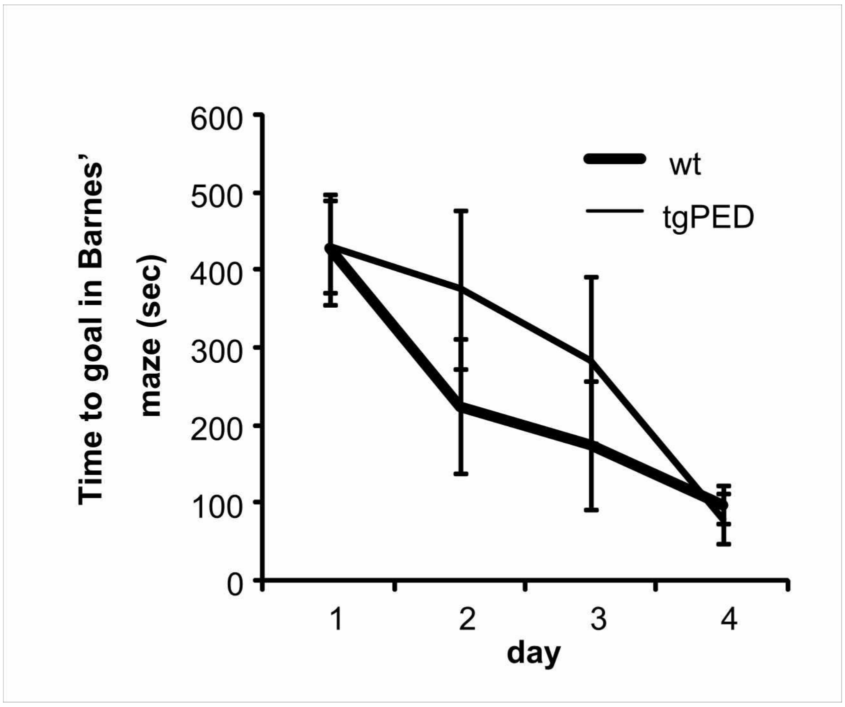

Supplementary Table 1. Behavioral phenotypization of tgPED mice

**Behavioral test**	**Wt (mean±SEM)**	**tgPED (mean±SEM)**	**p**
Grid climbing (time to climb)	95±14.6 sec	192±29sec	**p=0.0059**, t-test for non paired data
Negative geotaxis	8.5±2.1sec	15.8±3.2sec	**p=0.05**, t-test for non paired data
Làt maze – horizontal activity	83.7±9 num. of corner crossings	60±6.8 num. of corner crossings	**p=0.04**, t-test for non paired data)
Làt maze – vertical activity	56.5±4.2 num. of rearings	47.9±7.3 num. of rearings	p=0.29, t-test for non paired data
Gait: stride length	6.5±0.63cm	6.7±0.36cm	p=0.8, t-test for non paired data
Gait: stride width	2.6±0.17cm	2.3±0.1cm	p=0.15, t-test for non paired data
Beam walking 1st exposure	1.2±0.36 errors	2.9±0.6 errors	p=0.03, t-test for non-paired data
Beam walking, 3rd exposure	1.8±0.9 errors	1.37±0.72 errors	P=0.69; t-test for non paired data
Hanging grip test (Suppl table 1)	0.86±0.1N	0.85±0.07N	p=0.9, t-test for non paired data
Wire suspension test	13±4.9sec	15±1.9sec	p=0.7, t-test for non paired data
Vertical pole test	24.7±12.5sec	13.3±3.6sec	p=0.4, t-test for non paired data
O-maze – % time in unprotected arms	4.3±1.3 %	2.1±0.8 %	p=0.14, t-test for non paired data
O-maze – number of total entries in the open arms	2.5±1.2 entries	1±0.7 entries	p=0.2 , Mann-Whitney U test
O-maze – number of total entries in the closed arms	3.5±1.2 entries	2±0.7 entries	p=0.2, Mann-Whitney U test
EPM – % time in unprotected arms	10.1±3.7 %	3.8±1.9 %	p=0.14, t-test for non-paired data

EPM – Number of total entries in the open arms	3.3±1	1±0.7	**p=0.03**, Mann-Whitney U test
EPM – Number of total entries in the closed arms	10±1.6	4±1	**p=0.01**, Mann-Whitney U test
ORT – new object (% of exploration time: recognition index)	64.5 ±6.7 %	41.6±4.7 %	**p=0.012**, t-test for non-paired data
Rotarod - 1st exposure (time to fall, sec)	41.4±14.2sec	45.3±12.7	p=0.8, t-test for non-paired data
Rotarod - last exposure (time to fall, sec)	176.1±11.6	121.5±13.7	**p=0.008**, t-test for non paired data
Rotarod – slope of the learning curve	30±4.6	12.9±3.7	**p=0.03**, t-test for non paired data
Barnes maze - 1st exposure (time to goal, sec)	426.7±70.8	430±59.8	p=0.9, t-test for non paired data
Barnes maze- last exposure (time to goal, sec)	97.6±24.6	79.2±32.7	p=0.6, t-test for non paired data
Barnes maze – slope of the learning curve	-142.7±24.5	-94.1±29.8	p=0.2, t-test for non paired data
Barnes - working memory errors (number of holes visited two times in the same session)	5.2±2	4.9±1.2	p=0.86, Mann-Whitney
Barnes - procedural errors: number of visits to the goal without recognizing it	0.43±0.2	0.57±0.3	p=0.7, Mann-Whitney
Spontaneous alternation in T-maze (num. errors)	3.8±1	3.4±0.9	p=0.45, Mann-Whitney

Supplementary Table 2

Behavioral phenotype of four genetic models of PD (alpha-synuclein LRRK2, Parkin, PINK1, and DJ-1) compared to tgPED mice. Only models of familial forms of PD are presented. Most of the genetic PD models show motor and gait impairment (80%); memory impairment was described in 60% of models (due to lack of data), and anxiety in 20%. Data concerning glucose tolerance are also reported.

**Gene mouse model**	**Motor activity**	**Gait**	**Memory**	**Anxiety**	**Diabetes/Glucose tolerance**
Alpha-synuclein over-expression	Reduced [a]	Modified [l]	Memory impairment [a]	Increased [i]	Absent. Diet-induced glucose-intolerance accelerates the onset of symptoms [h]
LRRK2 ko	Normal [b] or mildly reduced [m]	Normal [b]	Untested	Normal [m]	Untested
PINK1 ko	Reduced[c]	Modified [d]	Untested	Untested	Improved glucose tolerance [g]
Parkin ko	Normal [e]	Modified [o]	Minor impariment [e]	Normal [n]	Untested
DJ-1 ko	Reduced [f]	Modified [q]	Memory impairment [f]	Untested	Greater susceptibility to diabetes [p]
tgPED	Reduced	Normal	Normal	Normal	Lower glucose tolerance

a Freichel C, Neumann M, Ballard T, Müller V, Woolley M, Ozmen L, Borroni E, Kretzschmar HA, Haass C, Spooren W, Kahle PJ. Age-dependent cognitive decline and amygdala pathology in alpha-synuclein transgenic mice. Neurobiol Aging. 2007 Sep;28(9):1421-35. Epub 2006 Jul 26.

b Hinkle KM, Yue M, Behrouz B, Dächsel JC, Lincoln SJ, Bowles EE, Beevers JE, Dugger B, Winner B, Prots I, Kent CB, Nishioka K, Lin WL, Dickson DW, Janus CJ, Farrer MJ, Melrose HL. LRRK2 knockout mice have an intact dopaminergic system but display alterations in exploratory and motor co-ordination behaviors. Mol Neurodegener. 2012 May 30;7:25. doi: 10.1186/1750-1326-7-25.

c Gispert S, Ricciardi F, Kurz A, Azizov M, Hoepken HH, Becker D, Voos W, Leuner K, Müller WE, Kudin AP, Kunz WS, Zimmermann A, Roeper J, Wenzel D, Jendrach M, García-Arencíbia M, Fernández-Ruiz J, Huber L, Rohrer H,

Barrera M, Reichert AS, Rüb U, Chen A, Nussbaum RL, Auburger G Parkinson phenotype in aged PINK1-deficient mice is accompanied by progressive mitochondrial dysfunction in absence of neurodegeneration. PLoS One. 2009 Jun 3;4(6):e5777. doi: 10.1371/journal.pone.0005777.

d Glasl L, Kloos K, Giesert F, Roethig A, Di Benedetto B, Kühn R, Zhang J, Hafen U, Zerle J, Hofmann A, de Angelis MH, Winklhofer KF, Hölter SM, Vogt Weisenhorn DM, Wurst W. Pink1-deficiency in mice impairs gait, olfaction and serotonergic innervation of the olfactory bulb. Exp Neurol. 2012 May;235(1):214-27. doi: 10.1016/j.expneurol.2012.01.002. Epub 2012 Jan 11.

e Zhu XR, Maskri L, Herold C, Bader V, Stichel CC, Güntürkün O, Lübbert H. Non-motor behavioural impairments in parkin-deficient mice. Eur J Neurosci. 2007 Oct;26(7):1902-11. Epub 2007 Sep 20.

f Pham TT, Giesert F, Röthig A, Floss T, Kallnik M, Weindl K, Hölter SM, Ahting U, Prokisch H, Becker L, Klopstock T, Hrabé de Angelis M, Beyer K, Görner K, Kahle PJ, Vogt Weisenhorn DM, Wurst W. DJ-1-deficient mice show less TH-positive neurons in the ventral tegmental area and exhibit non-motoric behavioural impairments. Genes Brain Behav. 2010 Apr;9(3):305-17. doi: 10.1111/j.1601-183X.2009.00559.x. Epub 2009 Dec 17.

g Deas E, Piipari K, Machhada A, Li A, Gutierrez-del-Arroyo A, Withers DJ, Wood NW, Abramov AY. PINK1 deficiency in b-cells increases basal insulin secretion and improves glucose tolerance in mice. Open Biol. 2014 May;4(5):140051.

h Rotermund C, Truckenmüller FM, Schell H, Kahle PJ. Diet-induced obesity accelerates the onset of terminal phenotypes in α-synuclein transgenic mice. J Neurochem. 2014 Dec;131(6):848-58. doi: 10.1111/jnc.12813. Epub 2014 Aug 11.

i Kim S, Park JM, Moon J3 Choi HJ. Alpha-synuclein interferes with cAMP/PKA-dependent upregulation of dopamine β-hydroxylase and is associated with abnormal adaptive responses to immobilization stress. Exp Neurol. 2014 Feb;252:63-74. doi: 10.1016/j.expneurol.2013.11.009. Epub 2013 Nov 16.

l Tatenhorst L, Eckermann K, Dambeck V, Fonseca-Ornelas L, Walle H, Lopes da Fonseca T, Koch JC, Becker S, Tönges L, Bähr M, Outeiro TF, Zweckstetter M2, Lingor P. Fasudil attenuates aggregation of α-synuclein in models of Parkinson's disease. Acta Neuropathol Commun. 2016 Apr 22;4:39. doi: 10.1186/s40478-016-0310-y.

m Bichler Z, Lim HC, Zeng L, Tan EK. Non-motor and motor features in LRRK2 transgenic mice. PLoS One. 2013 Jul 30;8(7):e70249. doi: 10.1371/journal.pone.0070249. Print 2013.

n Rial D, Castro AA, Machado N, Garção P, Gonçalves FQ, Silva HB, Tomé AR, Köfalvi A, Corti O, Raisman-Vozari R, Cunha RA, Prediger RD. Behavioral phenotyping of Parkin-deficient mice: looking for early preclinical features of Parkinson's disease. PLoS One. 2014 Dec 8;9(12):e114216. doi: 10.1371/journal.pone.0114216. eCollection 2014. Erratum in: PLoS One. 2015;10(3):e0118526.

o Navarro P, Guerrero R, Gallego E, Avila J, Luquin R, Garcia Ruiz PJ, Sanchez MP. Motor alterations are reduced in mice lacking the PARK2 gene in the presence of a human FTDP-17 mutant form of four-repeat tau. J Neurol Sci. 2008 Dec 15;275(1-2):139-44. doi: 10.1016/j.jns.2008.08.013. Epub 2008 Sep 24.

p Jain D, Weber G, Eberhard D, Mehana AE, Eglinger J, Welters A, Bartosinska B, Jeruschke K, Weiss J, Päth G, Ariga H, Seufert J, Lammert E. DJ-1 Protects Pancreatic Beta Cells from Cytokine- and Streptozotocin-Mediated Cell Death. PLoS One. 2015 Sep 30;10(9):e0138535. doi: 10.1371/journal.pone.0138535. eCollection 2015.

q Chandran JS, Lin X, Zapata A, Höke A, Shimoji M, Moore SO, Galloway MP, Laird FM, Wong PC, Price DL, Bailey KR, Crawley JN, Shippenberg T, Cai H. Progressive behavioral deficits in DJ-1-deficient mice are associated with normal nigrostriatal function. Neurobiol Dis. 2008 Mar;29(3):505-14. doi: 10.1016/j.nbd.2007.11.011. Epub 2007 Dec 4.
